# Serum Anti-Mullerian Hormone and Estradiol Concentrations in Gilts and Their Age at Puberty

**DOI:** 10.3390/ani10112189

**Published:** 2020-11-23

**Authors:** Nutthee Am-in, Junpen Suwimonteerabutr, Roy N. Kirkwood

**Affiliations:** 1 Department of Obstetrics, Gynecology and Reproduction, Faculty of Veterinary Science, Chulalongkorn University, Bangkok 10330, Thailand; nutthee.a@chula.ac.th (N.A.-i.); Junpen.S@chula.ac.th (J.S.); 2 Swine Reproduction Research Unit, Chulalongkorn University, Bangkok 10330, Thailand; 3 School of Animal and Veterinary Sciences, University of Adelaide, Roseworthy, SA 5371, Australia

**Keywords:** gilts, puberty, exogenous gonadotrophins, serum anti-Mullerian hormone

## Abstract

**Simple Summary:**

It has been documented in several species that blood levels of the hormone, anti-Mullerian hormone (AMH), are an indirect measure of the number of ovarian follicles and that the number of ovarian follicles may be an indication of potential female fertility. In the present study, we determined AMH levels at various ages in immature female pigs and related these to potential measures of future fertility including age at puberty and numbers of ovarian follicles at puberty, and the gilt response to ovarian stimulation after puberty. Our results support the suggestion that blood levels of AMH are reflective of future fertility in gilts.

**Abstract:**

For experiment one, blood samples were obtained from 200 gilts at 90, 120, 150, 180, and 200 days of age. Serum samples from the 30 youngest (166.1 ± 0.7 days) and 30 oldest (198.8 ± 0.6 days) gilts exhibiting estrus by 200 days, and a further 18 gilts that remained anestrus at 200 days, were assayed for serum concentrations of anti-Mullerian hormone (AMH) and estradiol (E2). Gilts younger at puberty had higher (*p* < 0.05) AMH levels than those older at puberty, and both groups had higher AMH levels than anestrus gilts (*p* < 0.05). Regardless of age, serum E2 was higher (*p* < 0.05) in gilts that achieved puberty than in gilts remaining anestrus. At spontaneous pubertal estrus detection, there was no effect of pubertal age on the number of preovulatory ovarian follicles. For experiment two, 152 prepubertal gilts received an intramuscular (IM) injection of 400 IU eCG plus 200 IU hCG and then received fence-line boar contact to detect estrus onset. Serum AMH concentrations were higher (*p* < 0.05) in the first 25 gilts to exhibit puberty than the last 28 gilts, with the first gilts also having more preovulatory follicles (*p* < 0.0001). Taken together, these data support an association between serum AMH concentrations and degree of physiological maturity and ovarian follicular development in gilts.

## 1. Introduction

An important measure of sow herd performance and thus potential farm profitability is the lifetime number of pigs produced per sow, a metric dependent on both the litter sizes delivered and the number of litters produced (i.e., parity at removal). Various management or environmental factors can affect the parity at removal, but a primary influence will be the quality of the gilts selected for the sow herd [1,2]. While selection criteria for robustness, and by extension potential longevity, are recognized, indicators for potential fertility are limited. As reviewed by Patterson and Foxcroft [2], management protocols can be implemented to hasten puberty onset in gilts and to identify gilts that are earlier maturing and therefore potentially more fertile. However, of greater benefit to the industry would be an indicator of future potential fertility determined at a younger age, prior to final selection of replacements.

It has been suggested that female fertility is influenced by various factors including the size of the ovarian follicular reserve [3,4]. The ovarian reserve is a term that refers to both the number of follicles in the follicular pool as well as their quality, with the age-associated decrease in women’s fertility being associated with a shrinking ovarian reserve [4]. There is now compelling evidence that a marker for the size of the ovarian reserve is anti-Mullerian hormone (AMH), which is produced only in granulosa cells of growing follicles in most species [3,5,6,7]. However, in pigs, AMH expression has also been detected in preovulatory ovarian follicular theca cells and in corpora lutea [8]. There is also limited evidence that serum AMH concentration in juvenile gilts at weaning are associated with improved estrus responses to later boar exposure [9], although there were no age-related differences in serum AMH concentration in 60, 80, and 100-day old gilts. Furthermore, in heifers, it was shown that higher AMH concentrations were associated with younger ages at puberty [10].

One of the suggested functions of AMH is to limit both initial and cyclic follicle recruitment by inhibiting FSH-induced aromatase and LH receptor expression [11]. By influencing aromatase activity, an effect on estrogen production can be assumed and, indeed, an inverse relationship between serum concentrations of estrogen and AMH has been documented [12,13]. With the aim of determining whether associations exist between serum levels of AMH and E2, and estrus responses in gilts, the present study was undertaken to test the hypothesis that circulating AMH levels are indicative of gilt reproductive potential as indicated by differences in age at puberty and responses to exogenous gonadotrophins.

## 2. Materials and Methods

These studies were approved by the Institutional Animal Care and Use Committee, Chulalongkorn University, Bangkok (approval number 1931033), and performed on a 600-sow commercial facility in Eastern Thailand.

For experiment one, 200 Landrace x Yorkshire gilts were selected at 90 day of age and a minimum of 25 kg body weight. From selection until detection of their first estrus, gilts were housed in groups of six in an open housing system allowing 2.0 m^2^ per head. Gilts were group-fed a diet formulated to provide 3200 kcal DE/kg and 15% crude protein allowing up to 2.5 kg/pig/day, with water supplied ad libitum from water nipples. From 150 to 200 days of age, gilts had direct exposure to mature boars for 15 min/day to stimulate onset and facilitate detection of their pubertal estrus. Blood samples were obtained from all gilts at 90, 120, 150, 180, and 200 days of age by jugular venipuncture and left to clot for 2 h at room temperature. The tubes were then centrifuged at 1000× *g* for 20 min and serum harvested and stored at −80 °C for less than two months. Serum from the 30 youngest and 30 oldest gilts exhibiting estrus by 200 days and 18 gilts that remained anestrus at 200 days were assayed for AMH and estradiol concentrations. At the detection of first their estrus, gilts were subject to ovarian examination to determine numbers of pre-ovulatory follicles (>6 mm) using transcutaneous real-time ultrasonography with a 5-MHz convex probe (HS-2000, Honda Electronics Co. Ltd., Tokyo, Japan).

For experiment two, 152 Landrace x Yorkshire gilts were managed as for experiment one. At approximately 165 days and 100 kg body weight, all gilts were blood sampled and samples processed as for experiment one for determination of progesterone to confirm non-cyclic status, and AMH. Then, all gilts received an intramuscular injection of 400 IU eCG plus 200 IU hCG (PG600^®^; Merck Animal Health, Summit, NJ, USA). Starting 2 days after injection, gilts were provided with daily fence-line contact with mature boars until day 10 to facilitate estrus detection. Gilts exhibiting estrus by 7 days were examined by ultrasound to determine numbers of pro-ovulatory follicles as described for experiment one.

Serum progesterone concentrations were determined by ELISA as described by Tummaruk et al. [14]. Assay sensitivity and intra- and inter-assay coefficients of variance (CV) were 0.06 ng/mL, 1.78%, and 2.14%, respectively. Serum estradiol concentrations were determined using a commercial kit (Estradiol Serum EIA Kit, Arbor Assays, MI, USA) at a 1:4 dilution and following the manufacturer’s instructions. The assay detection range was 3.75 to 120 pg/mL and intra- and inter-assay CV were 2.95 and 10.5%, respectively. Serum AMH concentrations were determined using a commercial kit (Porcine AMH ELISA, Ansh Lab, TX, USA) at a 1:2 dilution and following the manufacturer’s instructions. Samples with known concentrations of hormone were incorporated into the assay for quality control. The assay detection range was 0.2 to 12.0 ng/mL and the intra- and inter-assay CV were 3.67 and 9.82%, respectively.

### Statistical Analysis

All statistical comparisons employed SPSS (IBM SPSS statistics version 22.0.0.0 for Windows, SPSS Inc., Chicago, IL, USA). For experiment one, changes in serum concentrations of AMH and E2 with age were examined within the study group by repeated measure analysis of variance (ANOVA). To analyze the changes in more detail, at 90, 120, 150, 180 and 200 days of age, a pair *t*-test was performed as a post-hoc test. One-way ANOVA was used to explore differences between study groups within age, and Tukey’s was used as the post-hoc test. Correlations between the age at puberty onset and serum hormone concentrations were examined by means of the Pearson’s correlation test.

For experiment two, serum AMH concentrations and numbers of pre-ovulatory follicles were compared using the Student’s t-test for the first 25 gilts and last 30 gilts to exhibit estrus. For both experiments, the results are presented as mean ± SD and the limit of significance was set at *p* ≤ 0.05.

## 3. Results

For experiment one, the mean ages at puberty for the 30 youngest and 30 oldest gilts were 169.3 ± 0.6 day and 195.2 ± 0.9 day, respectively. As detailed in Table 1, serum AMH concentrations of the youngest group at 90, 120, and 150 days of age were higher than those of the oldest and anestrus groups (*p* < 0.05), but differences were not evident at 180 and 200 days of age. The anestrus gilts had lower serum AMH values than the youngest and oldest groups at all ages (*p* < 0.05; Table 1). There were significant negative correlations between age at puberty onset and serum AMH concentrations at 90, 120, and 150 days of age (Table 2).

In the youngest gilts, serum E2 concentrations were stable at 90 and 120 day, were significantly higher at 150 day, and then remained stable to 180 day (Table 1). A similar pattern was observed for the oldest gilts, although the increase was not evident until 180 days (Table 1), resulting in a significant correlation between age at puberty and day 150 serum E2 levels (Table 2). Compared to gilts achieving puberty, serum E2 concentrations were lower (*p* < 0.05) for anestrus gilts at all ages studied (Table 1). There was no gilt pubertal age effect for numbers of pre-ovulatory follicles; 16.5 ± 2.2 and 15.1 ± 0.64 for the youngest and oldest gilts, respectively.

**Figure 1:**
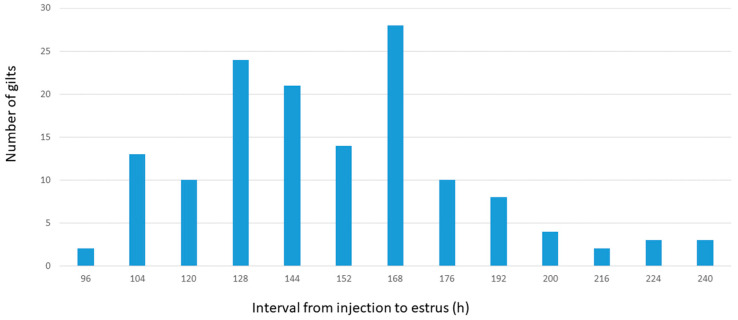
Distribution of intervals to estrus detection in gilts following injection of 400 IU eCG plus 200 IU hCG (PG600).

For experiment two, all gilts were deemed to be pre-pubertal at the start of the study. The first 25 gilts achieved puberty between 96 and 120 h and the last 30 gilts were pubertal between 176 and 240 h (Figure 1); 10 gilts remained anestrus. Mean serum AMH concentration in the first 25 gilts to exhibit estrus was higher (*p* < 0.02) than in the last 30 gilts (13.6 ± 1.2 and 11.8 ± 1.1 ng/mL). The first 25 gilts also had more (*p* < 0.001) pre-ovulatory follicles than the last 30 gilts (13.3 ± 1.3 and 11.4 ± 1.2).

## 4. Discussion

The present results demonstrated a negative association between serum AMH concentrations and age at puberty in gilts. Furthermore, it has been suggested that earlier maturing gilts are either innately more fertile or are able to undergo more prebreeding estrous cycles, resulting in improved fertility. In ruminants, a link between the size of the antral follicle pool and heifer fertility has been documented [15] but, unfortunately, there is a dearth of information regarding circulating AMH concentrations and ovarian morphology in gilts. It has also been noted that while aspects of immune localization of AMH in porcine ovaries are comparable to other species, differences do occur [8]. However, currently, it is reasonable to assume that circulating AMH concentration in prepubertal gilts are reflective of the size of the antral follicle pool. Therefore, by extension, our data suggest a link between prepubertal circulating AMH concentrations and subsequent gilt fertility. This concurs with earlier results from heifers, which showed that higher prepubertal AMH concentrations were associated with younger ages at puberty [10]; however, species differences were apparent. The latter authors documented an initial increase in circulating AMH peaking at 10 weeks of age and thereafter declining to plateau from about six weeks prior to puberty. They suggested that this initial increase was due to an increase in antral follicle counts in calves between two and four months of age, and the number of antral follicles were associated with earlier calving dates [15].

The reaching of an AMH plateau prior to puberty in heifers presumably reflects the achievement of an adult type ovary where continued waves of follicle recruitment would result in an unchanging AMH level. In contrast, our data indicate that in our relatively earlier maturing gilts, circulating AMH levels remained essentially unchanged between 90 and 200 day of age. This lack of gilt age-related change in circulating AMH supports previously published data that documented no evident difference in gilt circulating AMH between 60 and 180 day [12] or at 80 vs. 160 day [13].

In the later maturing gilts in our study, serum AMH was lower than in the earlier maturing gilts until levels increased from 180 day. A broadly similar pattern was observed for serum E2 concentration in that the gilts achieving puberty at younger ages demonstrated an E2 elevation from 150 day whereas, in the older maturing gilts, an elevation was noted at 180 day. Of interest, these increases in serum E2 occurred at similar intervals before the mean ages at puberty. Furthermore, the increased serum E2 detected at 180 day was associated with an increase in AMH to concentrations noted in the earlier maturing gilts. This may suggest that the inhibitory effects of AMH on FSH activity [11], and so by extension E2 production, declines in the gilt peripubertal period. Although speculative, it seems that some as yet undefined permissive degree of ovarian maturity must be achieved before puberty can occur. Regardless, the number of preovulatory ovarian follicles was not influenced by age at puberty. The uniformly lower circulating AMH and E2 concentrations in gilts destined to remain anestrus at 200 day of age suggests that they likely had smaller antral follicle pools, which resulted in a failure to achieve levels of ovarian maturity necessary to initiate estrous cycles.

The data from experiment two support the association between circulating AMH concentrations and ovarian activity. Injection of 400 IU eCG and 200 IU hCG into gilts at 165 day of age would be expected to induce estrus. By commercial standards, the estrus response was very good in 142 of the 152 treated gilts (93.4%) exhibiting behavioral estrus. However, the intervals from injection to estrus detection were variable and influenced by circulating AMH concentrations; the most rapidly responding gilts had higher AMH levels. This indicates that higher AMH levels are associated with a more responsive ovarian follicle pool, a suggestion supported by the increased number of preovulatory follicles observed at estrus detection in the gilts with the shortest injection to estrus intervals. The impact on gilt fertility of higher serum AMH and increased ovarian response to exogenous stimulation remains to be determined.

## 5. Conclusions

Our data support the hypothesis that circulating AMH levels are indicative of gilt reproductive potential as indicated by differences in age at spontaneous puberty and estrus and ovulatory responses to exogenous gonadotrophins. These data relate only to the study population so generalization of a targeted selection threshold is not appropriate. Further work is needed across genetics, herds, and management to develop this measure. However, if successful, its’ application would be invaluable to the global swine industry.

## Figures and Tables

**Table 1:** Gilt serum concentrations of anti-Mullerian hormone (AMH) and E2 at 90, 120, 150, 180, and 200 days of age.

Serum Hormone Levels	Age (days)	Youngest (*n* = 30)	Oldest (*n* = 30)	Anestrus (*n* = 18)
AMH (ng/mL)	90	12.5 ± 1.1 ^a,x^	9.8 ± 1.2 ^b,x^	7.6 ± 1.2 ^c,x^
	120	12.6 ± 1.2 ^a,x^	9.8 ± 1.1 ^b,x^	7.9 ± 1.1 ^c,x^
	150	12.9 ± 1.1 ^a,x^	10.1 ± 1.1 ^b,xy^	8.4 ± 1.1 ^c,x^
	180	13.1 ± 1.3 ^a,x^	12.8 ± 1.2 ^a,xy^	9.1 ± 1.2 ^b,x^
	200	13.8 ± 1.2 ^a,x^	13.0 ± 1.4 ^a,y^	9.1 ± 1.4 ^b,x^
E2 (pmol/L)	90	95.4 ± 11.2 ^a,x^	89.2 ± 8.3 ^a,x^	65.2 ± 6.2 ^b,x^
	120	100.2 ± 8.5 ^a,x^	94.5 ± 5.2 ^a,x^	68.4 ± 9.1 ^b,x^
	150	122.3 ± 12.1 ^a,y^	95.4 ± 8.1 ^b,x^	69.3 ± 11.2 ^c,x^
	180	130.2 ± 9.5 ^a,y^	124 ± 10.2 ^a,y^	70.6 ± 6.7 ^b,x^
	200	129.4 ± 14.2 ^a,y^	126.5 ± 11.2 ^a,y^	72.8 ± 9.2 ^b,x^

^a,b,c^ Rows with different superscripts differ *p* ≤ 0.05; ^x,y^ Columns with different superscripts differ *p* ≤ 0.05.

**Table 2:** Correlations between the age at puberty onset and hormone serum levels at 90, 120, 150, 180, and 200 days of age (*n* = 60).

Serum Hormone Levels	Age (Days)	r	*p* Value
AMH (ng/mL)	90	−0.81	0.005
	120	−0.67	0.002
	150	−0.64	0.001
	180	−0.41	0.07
	200	−0.32	0.12
E2 (pmol/L)	90	0.31	0.18
	120	0.41	0.09
	150	−0.74	0.02
	180	0.32	0.16
	200	−0.51	0.08
